# Cross-cultural study of kinship premium and social discounting of generosity

**DOI:** 10.3389/fpsyg.2023.1087979

**Published:** 2023-02-24

**Authors:** Jiawei Liu, Edmund Derrington, Julien Bénistant, Brice Corgnet, Jean-Baptiste Van der Henst, Zixuan Tang, Chen Qu, Jean-Claude Dreher

**Affiliations:** ^1^Laboratory of Brain, Cognition and Education Sciences, Ministry of Education, Guangzhou, China; ^2^Guangdong Key Laboratory of Mental Health and Cognitive Science, School of Psychology, Center for Studies of Psychological Application, South China Normal University, Guangzhou, China; ^3^Laboratory of Neuroeconomics, Institut des Sciences Cognitives Marc Jeannerod, CNRS, Lyon, France; ^4^UFR Biosciences, Université Claude Bernard Lyon 1, Lyon, France; ^5^EmLyon, Écully, France

**Keywords:** social discounting, kinship premium, altruism, cultural collectivism, Chinese, generosity

## Abstract

Social discounting predicts that one’s concern for others decreases with increasing social distance. Cultural dimensions may influence this social behavior. Here, we used a dictator game, in which the participants and real members of their social entourage profited from the partition of the endowments determined by the participant, to compare how Chinese and French university students shared endowments with people at different social distances. We tested two hypotheses based on the concepts of kinship premium and cultural collectivism. Stronger ties between close family members were expected among Chinese. This may predict a larger “kinship premium,” i.e., increased generosity to family members at close social distances, in Chinese relative to French participants. Similarly, because collectivism is thought to be stronger in Asian than western societies, greater generosity at larger social distances might also be expected among Chinese participants. The results showed that Chinese were more generous than French at close social distances but discounted more as social distance increased. This difference between French and Chinese was confined to family members and no significant difference in generosity was observed between French and Chinese for non-family members at any social distance. Our findings evidence a stronger kinship premium among Chinese than French students, and no significant effect of cultural collectivism.

## 1. Introduction

Altruism, generosity at one’s own expense, is an important component of human prosocial behavior. However, individuals are not equally generous to everyone and tend to favor family and people with whom they have close personal relationships. Social discounting is based on the concept of temporal discounting, a well characterized phenomenon by which a reward progressively loses subjective value with the delay that is imposed prior to the reward’s arrival (Rachlin et al., 1991). In either real or hypothetical experiments, participants have been asked to choose how to allocate money or other resources, between themselves and potential recipients at different social distances. The amount donated usually falls with increasing social distance between the two parties (Wu et al., 2011; Strang et al., 2017). Thus, the subjective value of being more generous is discounted as social distance increases.

One of the most studied inter-cultural dimensions is the degree of Collectivism/Individualism (Hofstede, 1980b,^1984^; Triandis, 1989). Collectivism is a characteristic of cultures in which people perceive themselves to be integrated and interdependent, whereas in individualistic societies, people are more focused on their own wellbeing, and that of closely related others such as immediate family (Hofstede, 1980a, p. 45; Hofstede, 1984; Markus and Kitayama, 1991; Kitayama and Uskul, 2011). One might thus expect different patterns of social discounting in participants who conform to collectivist as opposed to individualist cultural identities. Cross-cultural variations in social discounting might even provide an objective measure for the degree of social integration of individuals with other members of their society.

Generosity to genetically related others, such as close family members, has also been distinguished from generosity to friends or other unrelated individuals (Darlington, 1978). Indeed, more recently, studies have shown that kinship creates a specific unique contribution to generosity over and above the effects of emotional closeness such that participants in experiments are more generous to kin than might be expected if generosity depended on emotional closeness alone. This has been defined as “kinship premium” (Curry et al., 2013; Pollet et al., 2013; Booysen et al., 2018a). Social discounting has also evidenced such kinship premiums (Rachlin and Jones, 2008; Booysen et al., 2018a).

Collectivism or social interdependence is thought to be stronger in Asian than in western societies (Leung and Iwawaki, 1988; Hui et al., 1991; Oyserman et al., 2002). Previous cross cultural studies have compared social discounting in Japanese and German students (Ishii and Eisen, 2018), Chinese and German students (Strombach et al., 2014) and Japanese and American students (Ito et al., 2011; Romanowich and Igaki, 2017; Stegall et al., 2019). Two studies found that western participants were more generous at close social distances but discounted more rapidly as social distance increased, and this was taken as evidence that the greater social cohesiveness of Asian societies was reflected by lower levels of social discounting (Strombach et al., 2014; Ishii and Eisen, 2018). However, two studies reported no significant differences between Asian and Western participants (Romanowich and Igaki, 2017; Stegall et al., 2019) or Americans and Germans (Stegall et al., 2019), and one found that Asian participants (Japanese), discounted more rapidly than American college students as social distance increased (Ito et al., 2011). These mixed findings indicate that more research is needed to understand the influence of cultural differences on social discounting, and, no study analyzed whether social discounting was affected by cross-cultural differences in kinship premiums.

Most cross-cultural studies used abstract games to study social discounting. All were based on binary decision tasks that measure participants’ preference for a generous or a more selfish option, to divide money or other resources between themselves and another at a given social distance. This forced choice may influence the decisions made by the participants. Indeed, neither of the options offered on a given trial may reflect the true preference of the participants. Furthermore, calculation of the participants’ generosity at a given social distance can be complicated because an accurate estimation of a participant’s generosity depends on the participant behaving consistently across all the trials at each social distance. This is frequently not the case (Booysen et al., 2018b). Finally, many of the previous studies are completely or partially hypothetical, with the participants’ decisions having no concrete consequences for themselves and/or the hypothetical “recipients.”

We took a new approach to study social integration and kinship premium by analyzing social discounting with a concrete dictator game. Participants chose freely how to divide endowments, that varied in size on different trials, with recipients at different social distances. These recipients were not hypothetical, participants identified them from their own social entourage. Only at high social distances were potential beneficiaries anonymous. At the end of the experiment a single trial was chosen at random and its consequences applied to both the participant and the recipient. Thus, both participants and recipients would enjoy the fruits of the participants’ avarice or largesse. This non-deceptive and non-hypothetical approach allowed us to mitigate concerns that have been raised concerning hypothetical biases that occur in similar economic games (Forsythe et al., 1994; Ben-Ner et al., 2008; Bühren and Kundt, 2015). Furthermore, participants were absolutely free to choose how much to donate, without any external cues as to what might constitute a generous or selfish allocation. Finally, we used participants from two countries, France and China, known to exhibit different levels of Individualism/Collectivism. This rigorous behavioral economics approach allowed us to study first whether social discounting differs between groups with different levels of Collectivism and second to compare kinship premiums in university students from the two countries.

## 2. Materials and methods

### 2.1. Participants

A total of 102 subjects participated in our experiment. Testing was carried out in 2 laboratories: South China Normal University (Guangzhou, China) and Institute of Cognitive Science (Lyon, France). A total of 51 Chinese participants (26 females; *M _age_* = 20.45, *SD* = 1.36) were students recruited at the SCNl University and 51 French participants (25 females; *M _age_* = 21.24, *SD* = 2.14) were students recruited from the Lyon 1 University. The sample size was based on a reference study in the domain which tested around 50 participants per group (Strombach et al., 2014). All participants were psychiatrically and neurologically healthy and indicated they were not taking any medication. The two studies were approved by the local ethics committees. All experimental protocols and procedures were conducted in accordance with institutional review-board guidelines for experimental testing and complied with the latest revision of the Declaration of Helsinki. Participants gave written consent for participation in the study and were free to leave at any time.

### 2.2. Experimental design

Subjects were required to perform a self-representation task in which they rated their level of intimacy with a list of relationships on a 100-point scale: mother, father, siblings, grandparents, kin, best friend, roommate, colleagues, neighbors, acquaintances, partner, strangers and friend circle. When subjects had no equivalent social relationship (e.g., partner) they skipped that question.

Subjects then performed the social discounting task, which was a dictator game repeated over 40 trials in a random order in which subjects chose how to split an endowment between themselves and identified recipients at different social distances. First, the subjects were required to identify the recipients from their own social entourage that best corresponded to each social distance (1, 2, 3, 5, 10, 20, 50, and 100), and indicate the relationship between them (i.e., Social Distance 1, name: Mary March, relationship: Mother). Participants were informed that a random trial would be selected, in which the receiver was sent 10% of the amount indicated by the participant for that trial. Importantly, because by definition the receivers at social distances 50 and 100 corresponded to someone that participants did not know, the money for the trials corresponding to these social distances would be sent to a charity project (online payment platform). We also varied the size of the endowment within subjects with five different endowment sizes at each social distance (€80, €90, €100, €110, and €120, or the same amount in Yuan for Chinese subjects). This allowed as to calculate mean values for generosity at each social distance for each participant and to determine whether the endowment size altered their generosity.

**FIGURE 1:**
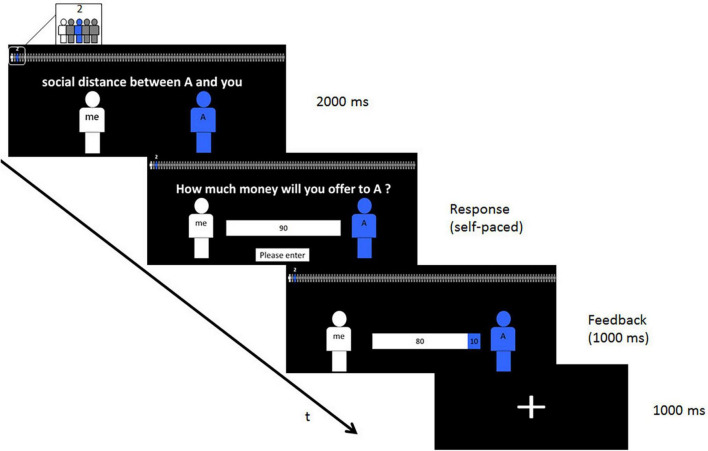
Experimental design. After a fixation cross, a social distance line was displayed across the top of the screen showing a white and a blue person, respectively, representing the participant and the receiver. The number above the blue icon indicated their exact social distance to the participant. Then, participants were shown one of 5 possible endowments (€80, €90, €100, €110, €120 for French, or the same amount in Yuan for Chinese subjects). Participants had to type on a keyboard the exact amount that they were willing to send to the recipient (here €10). A feedback screen indicated the amounts for the participant (here €80), and the receiver (here €10).

In each trial, social distance was represented on a scale of 101 small icons displayed at the top of the screen. The subject was indicated by a white icon at the left extremity, and the receiver by a blue icon labeled with a number, the smaller the number, the closer the social distance (Figure 1). Unlike previous studies, which used forced choices, in our study generosity could be measured directly as the proportion of the endowment that the participants chose freely to donate. The amount of the endowment for the trial was displayed on the screen, and the participant indicated how much was given to the recipient. This amount could not exceed the endowment. Finally, at the end of the trial the participant was reminded how the money had been allocated.

Participants received a fixed amount of € 6 in France and 10 Yuan in China. These amounts were selected because in July 2016 (when the experiments took place), the French purchasing power was about 1.6 times that in China. In addition, the participant received their share of the allocation from the randomly selected trial paid at 10% of its full value. Participants transferred the money directly to their recipients under the supervision of experimenters by bank accounts (French), and by the payment application of Alipay or Wechat (Chinese).

Once the 40 trials elapsed, participants completed the shortened version of the Individualism-Collectivism (IND-COL) scale composed of 33 items on a 6-point scale to test for between and within culture variations (Hui and Yee, 1994). This scale allows participants to be scored with respect to five primary categories of Individualism and Collectivism: “Kin and neighbors/susceptibility to influence (KN),” measures the extent to which participants are influenced by family and neighbors as opposed to having a more independent attitude, “Neighbor/social isolation (NE),” measures the extent of social interaction and casual relationships with their neighbors, “Colleagues and friends/supportive exchanges (CF)”: measures the degree of interdependence of participants with coworkers and friends, “Parents and spouse/distinctiveness of personal identity (PS),” measures the participants’ individualism within their nuclear family, and, “Parents/consultation and sharing (PA)”: measures the extent to which participants are influenced by and interact with their parents. Two second order factors, “Ingroup Solidarity” (calculated from the sums of the CF, PA, and PS subscales) and “Social Obligation” (calculated from the sum of the KN and NE subscales) and a global IND-COL score (the sum of all five subscales) are calculated from these scores. Finally, participants were asked to what extent they identified with their own culture on a scale from 1 to 5.

The task was programmed using *E-prime 2.0* and all the instructions were delivered in the subjects’ native language. The detailed instructions, can be found in the Supplementary material section.

### 2.3. Model estimation

To analyze social discounting we fitted the following discounting functions: the Constant Sensitivity model [Ebert and Prelec, 2007; Eq. (1)], Beta-Delta model [Laibson, 1997; Eq. (2)] and the Hyperbolic model [Jones and Rachlin, 2006, 2009; Eq. (3)]. One of our central hypotheses was that individuals with different levels of collectivism/individualism may show different patterns of social discounting that may be best accounted for by different models, or by the same model but with different values of the pertinent variables for that model.


(1) $$v=e^{\left[-{\left(a*D\right)}^{b}\right]}$$

(2) $$v=\beta *\delta^{D}$$

(3) $$v=\frac{V}{1+k\,D}$$

In all the equations v indicates the subjective value to the participant of being generous to the recipient and D symbolizes the social distance. Respectively, Eq. (1), the Constant Sensitivity model: *a* sets the distance to a theoretical boundary that separates close from distant relationships, and *b* represents the degree of sensitivity to social distance. For smaller values of *b* there is less sensitivity to social distance, which indicates that the participant discriminates less between recipients at closer and recipients at more distant social distances. Eq. (2), the Beta-Delta model: β represents the extra value awarded to the closest social distance value and δ captures a constant rate of social discounting, that is, δdetermines the rate at which generosity decreases as social distance increases, in the standard exponential model. When δ is higher the participants will discriminate proportionally more between recipients at closer and more distant social distances Eq. (3), the Hyperbolic model: *k* represents a constant that measures the degree of social discounting, i.e., the extent to which generosity decreases at each social distance compared to *V*, which is the theoretical undiscounted value of generosity at distance *D* = 0. Higher values of *k* will result in the participants discriminating proportionally more between recipients at closer and recipients at greater social distances.

We fitted the models to the individual social discounting curves of each participant. The curves were generated from the mean proportions of each of the five endowments allocated by each participant, at each social distance. Use of the mean percentage of the amount given by each participant, as opposed to the median was justified by the fact that our participants made free choices, as opposed to forced choices between a series of more or less generous offers at each social distance (Rachlin and Jones, 2008; Strombach et al., 2014). The non-linear least-squares estimation, *nlmrt* package (Nash, 2012) in R was used to calculate the Akaike Information Criterion (AIC) for how well each individual fit each model, and the sum of AIC for all individuals fitting that model was used to calculate the group AIC. Since the overwhelming majority of AIC were negative (98.5%), the sum of the AIC for all the subjects fitting that model reflects the goodness of fit of the whole group of curves, one for each participant, to each model. The model with the lowest Group AIC was that which best reflects the group as a whole. The mean Individual AIC reflects how well each individual in the Group fits each model. Results from the model estimation show that one Chinese and one French participant did not fit the Hyperbolic model.

### 2.4. Regression analysis

To address how specific factors (e.g., Culture, family relationships, subscales of IND-COL scale etc.) affected generosity across social distance, we conducted 6 separate generalized linear models (GLMs) with the proportion of the endowment given as a dependent variable. We use GLMs with a logit link function and a binomial family to account for the specificity of the dependent variable, we also clustered the standard error at the participant level. Specifically, we ran 3 GLMs at social distances 1∼100 (GLMs 1, 2, and 3), and 3 GLMs for social distances 1∼20 (for GLMs 4, 5, and 6), using the glm function from STATA. We included in all GLMs the following independent variables: whether the participant was French or Chinese (variable French, Chinese = 0, French = 1), the social distance (8 level values for GLMs 1, 2, and 3: 1, 2, 3, 5, 10, 20, 50, and 100; and 6 values for GLMs 4, 5, and 6: 1, 2, 3, 5, 10, and 20), the endowment size (€80, €90, €100, €110, €120), whether the recipient was a family member or not (variable Family member). In GLMs 2 and 5, we also included interactions between the variable Family member, Distance and French. In addition to these interactions, we also included the five subscales of the IND-COL as variables in GLMs 3 and 6.

To identify correlations between social discounting and the participants’ scores on the IND-COL scales, linear regressions were performed between the participants’ IND-COL scores on each of the five subscales, the 2 s order factors (Ingroup solidarity and Social Obligation) and their global IND-COL score with their individual scores for the parameters in the three models (*a* and *b* for the Constant Sensitivity model, β and θ for the Beta-Delta model and V and k for the hyperbolic model) in R.

## 3. Results

### 3.1. Cultural comparison of French and Chinese participants

Participants rated their level of identification with their respective culture on a five-point Likert scale. Chinese participants felt significantly more connected to their culture than the French, (Mann-Witney *U*-test; M Chinese = 4.08, SD Chinese = 0.72; M French = 3.67, SD French = 0.91; *z* = 2.58, *p* = 0.01, *U* = 1648). This closer association was similar to that reported previously when Chinese were compared to Germans (Strombach et al., 2014).

For the Self-Representation task, the Chinese self-rated their closeness to progenitors (mother, father, grandparents) and groups of people in society (neighbors, circle of friends, acquaintances, or strangers) significantly closer than the French (unpaired *t*-test, *p* < 0.05) (Table 1). However, Chinese and French participants were not different in their ratings of closeness to their peers (siblings, best friend, roommates, colleagues or partner). Globally these results show a tendency for French participants to consider themselves to be more socially distant than the Chinese from family authority figures (parents and grandparents), and also groups of people in society (circle of friends, neighbors, strangers). This is broadly in agreement with similar experiments comparing Chinese and German students (Strombach et al., 2014). However, unlike German students, the French students in our study also considered themselves as more socially distant from strangers, their circle of friends, their neighbors and indeed their acquaintances than the Chinese.

**TABLE 1:** Mean perceived social distance in the self-representation task.

Relationship	Chinese	French	t	df	*p*
Mother	5.54 (9.39)	10.29 (10.52)	-2.397	98.14	0.018*
Father	6.53 (9.15)	20.08 (24.98)	-3.637	63.191	0.001**
Brother and sister	9.22 (11.08)	11.88 (13.24)	-1.097	96.629	0.275
Grandparents	14.80 (14.96)	26.75 (21.59)	-3.228	89.253	0.002**
Kinships	29.39 (21.59)	29.59 (18.96)	-0.049	98.359	0.961
Best friend	12.06 (10.32)	13.37 (13.31)	-0.557	94.144	0.579
Roommates	24.86 (16.95)	29.55 (22.15)	-1.2	93.615	0.233
Classmates	38.73 (21.32)	41.94 (22.26)	-0.745	99.817	0.458
Neighbors	47.29 (24.48)	71.41 (22.32)	-5.2	99.156	0.000***
Acquaintances	36.49 (21.49)	60.35 (21.60)	-5.593	99.997	0.000***
Partner	13.22 (19.11)	7.51 14.66)	1.274	34.166	0.211
Strangers	81.68 (19.75)	98.20 (5.54)	-5.697	56.529	0.000***
Circle of friends	47.98 (25.98)	72.00 (17.86)	-5.405	86.683	0.000***

The mean social distance to different types of social relations as estimated by the participants in the self-representation task. Standard errors are in parentheses. The significance of differences was determined by an unpaired student’s *t*-test. *p < 0.05, **p < 0.01, ***p < 0.001.

The shortened version of the Individualism-Collectivism scale (Hui and Yee, 1994), in which higher scores indicate more collectivist tendencies, also highlighted cultural differences (Table 2). Although there was no significant difference between the global IND-COL scores of the French and Chinese participants (*M*_Chinese_ = 12.82, *SD*_Chinese_ = 13.04; *M*_French_ = 9.35, *SD*_French_ = 12.12), Chinese participants scored significantly higher in two individual subscales, among five that represent how individuals relate to relationships. Specifically, the Chinese scored significantly higher than the French in the “Kin and neighbors/susceptibility to influence” (M Chinese = 1.10, SD Chinese = 5.58; M French = −0.94, SD French = 3.54; *t* = 2.204, *p* = 0.03) and the “Neighbor/social isolation” factors (M Chinese = −12.39, SD Chinese = 5.10; M French = −15.90, SD French = 5.14; *t* = 3.463, *p* < 0.001). For the three other individual subscales scores were not significantly different between the two pools of participants (Table 2). With regard to the higher order factors, Ingroup Solidarity, provides a measure of the participants collectivism with regard to the nuclear family and close friends, whereas, Social Obligation measures integration and collectivism with respect to society as a whole (Hui and Yee, 1994). There was no significant difference between the French and the Chinese students with respect to the former, however, for the latter the Chinese participants scored significantly higher (M Chinese = −11.29, SD Chinese = 7.91; M French = −16.84, SD French = 6.44; *t* = 3.885, *p* < 0.001), indicating a higher degree of collectivism with society as a whole.

**TABLE 2:** Results of individualism-collectivism scale IND-COL scales and subscales (Hui and Yee, 1994).

Scale	Chinese	French	t	df	*p*
CF	21.67 (5.25)	22.45 (4.01)	-0.848	93.502	0.398
PA	2.29 (2.68)	1.98 (2.79)	0.579	99.835	0.564
PS	0.37 (3.58)	1.76 (5.41)	-1.533	86.825	0.129
KN	1.10 (5.58)	−0.94 (3.54)	2.204	84.54	0.03*
NE	−12.39 (5.10)	−15.90 (5.14)	3.463	99.993	0.001***
In group solidarity	24.12 (8.97)	26.20 (8.16)	-1.224	99.12	0.224
Social obligation	−11.29 (7.91)	–16.84 (6.44)	3.885	96.031	0***
Global	12.82 (13.43)	9.35 (12.12)	1.37	98.972	0.174

Mean results of the IND-COL test for the Chinese and French students. Standard errors are in parentheses. The significance of differences was determined by an unpaired student’s *t*-test. *p < 0.05, ***p < 0.001. CF, PA, PS, KN, and NE stand for subscales colleagues and friends/supportive exchanges, parents/consultation and sharing, parents and spouse/distinctiveness of personal identity, Kin and neighbors/susceptibility to influence, and neighbor/social isolation, respectively. Higher scores indicate a greater degree of collectivism.

These results reinforce the picture that the Chinese students feel generally more integrated with their society as a whole than the French, however, differences with respect to close family and friends are less consistent. The French family relationships and in particular parents were scored as being significantly more distant than the Chinese on the self-representation task, but were not significantly different to the Chinese on the associated IND-COL subscales or with respect to Ingroup Solidarity.

### 3.2. Comparison of French and Chinese recipients at different social distances

Participants were required to choose recipients from their own social entourage (family and friends etc.) that best corresponded to the social distances 1, 2, 3, 5, 10, and 20, in which 1 corresponds to the closest person to them and 100 corresponds to a perfect stranger. The recipients at distance 50 and 100 were anonymous since the former would correspond to a person they occasionally encounter, but do not know, and the latter to a person they do not know at all.

**FIGURE 2:**
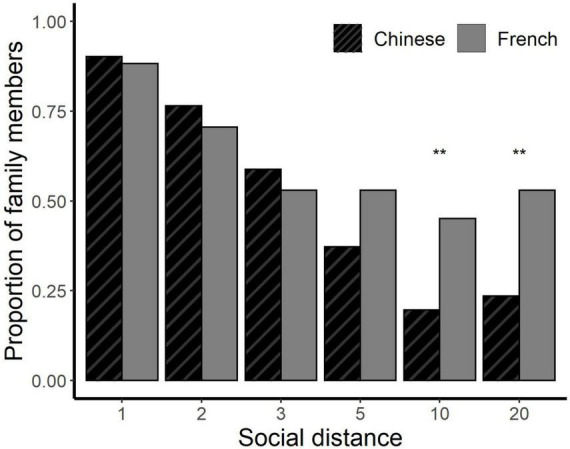
Inclusion of family at the social distance range 1∼20. Less participants choose family members as recipients at greater social distances, this decline was more rapid for the Chinese than for the French. *p < 0.05, **p < 0.01, ***p < 0.001.

We compared the types of relationship between Chinese and French participants and their choices for recipients at social distances 1 to 20. Both the French and the Chinese identified similarly high proportions of family members (Mother, Father, Brother or Sister, Grandparents, other blood relatives, and Partner) at social distances 1 (>90%), 2 (>70%), 3 (>50%) and 5 (>40%) (Figure 2). However, at social distances 10 and 20 about 40% of French participants continued to include blood relatives whereas significantly fewer Chinese participants, (about 20%), did so (social distance 10: (N Chinese = 10, N French = 23; χ^2^ = 6.451, df = 1, *p* = 0.011); and social distance 20: (N Chinese = 11, N French = 27; χ^2^ = 8.137, df = 1, *p* = 0.004).

For each social distance, we compared the proportion of the endowments donated across Groups (Table 3). As expected, generosity decreased as social distance increased. We observed a significant difference in the allocation of the endowment by the French and the Chinese at social distance 1, (M Chinese = 0.843, SD = 0.198; M French = 0.672, SD = 0.217, *t* = 4.15, *p* < 0.001); social distance 2, (M Chinese = 0.703, SD = 0.227; M French = 0.573, SD = 0.240, *t* = 2.81, *p* < 0.01); and social distance 3 (M Chinese = 0.165, SD = 0.131; M French = 0.114, SD = 0.153, *t* = 2.00, *p* < 0.05). However, at greater social distances there was no significant difference between the proportions of the endowments donated by the French and the Chinese.

**TABLE 3:** Proportion of the endowments allocated to recipients at each social distance.

Social distance	Chinese mean (SD)	French mean (SD)	t	df	*p*
1	0.84 (0.20)	0.67 (0.22)	4.147	99.211	0.000***
2	0.70 (0.23)	0.57 (0.24)	2.805	99.688	0.006**
3	0.66 (0.26)	0.56 (0.25)	2.001	99.978	0.048*
5	0.56 (0.27)	0.47 (0.20)	1.764	92.056	0.081
10	0.43 (0.24)	0.43 (0.21)	-0.036	97.477	0.971
20	0.36 (0.22)	0.36 (0.19)	0.097	98.502	0.923
50	0.17 (0.13)	0.11 (0.15)	1.831	97.719	0.070
100	0.07 (0.08)	0.08 (0.15)	-0.453	77.43	0.652

Standard errors are in parentheses; *p < 0.05, **p < 0.01, ***p < 0.001.

### 3.3. Model-based results

To determine how social distance affects generosity, we compared the group giving behavior of Chinese and French with 3 different models (the Hyperbolic model, the Beta-delta model, and the Constant Sensitivity model). The major difference between the three models concerns the predicted evolution of discounting behavior as social distance increases. All the models predict that generosity will decline as social distance increases. For the hyperbolic model and the Beta-Delta model the difference in generosity per unit of social distance should decline as social distance increases according to hyperbolic and exponential curves, respectively. Similarly, for the Constant Sensitivity model when *b* < 1 the generosity per unit of social distance declines exponentially as social distance increases. However, when *b* > 1 and social distance <1/a, generosity declines less rapidly per unit of social distance.

**FIGURE 3:**
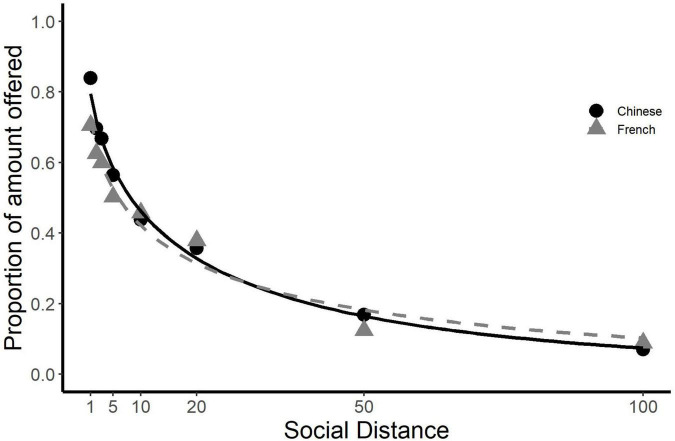
Social discounting behavior of Chinese and French participants in the Constant Sensitivity model. The graph shows the proportions of endowments offered by the participants to recipients at each social distance, as estimated by the Constant Sensitivity model, which was globally the best model to account for social discounting for all participants and for the Chinese participants in particular. Although it was not the best model of the French students’ discounting behavior, it fit them similarly well as the Chinese.

The best model to account for the data of Chinese participants was the Constant Sensitivity model (mean AIC Constant Sensitivity _Chinese_ = −64.95, Table 4 and Figure 3). Both the Hyperbolic model (mean AIC Hyperbolic _Chinese_ = −61.28) and the Beta Delta model (mean AIC Beta/Delta _Chinese_ = −59.79) were significantly worse at explaining their behavior. The best model to account for the data of the French participants was the Beta-Delta model (mean AIC Beta/Delta _French_ = −67.53). At the individual level this was only just better than the Constant Sensitivity model (AIC Constant Sensitivity _French_ = −65.48) and the Hyperbolic model (AIC Hyperbolic _French_ = −65.28), however, the above are the mean differences in AIC for each individual in the group. The Group differences in AIC are 51-fold higher (*N* = 51), indicating that the best models to account for the data of each group is much better than the second or third best models. Thus, although there is some justification for comparing the two groups with respect to the parameters *a* and *b* of the Constant Sensitivity model, which fits the French at least as well as it fits the Chinese, and is globally the best model for the two groups combined (Table 5), comparisons for the parameters of the Beta Delta model or the Hyperbolic model are rather more tenuous.

**TABLE 4:** Model selection according to AIC.

Model	Formula	Individual AIC (mean)		Group AIC	
		Chinese	French	Chinese	French
Constant sensitivity	*v* = *e*^[−(a^*^D)*^b^*]^	−64.95	−65.48	−3208	−3339
Beta-delta	*v* = β*δ^D^	−59.79	−67.53	−3049	−3444
Hyperbolic	$v=\frac{V}{1+k\,D}$	−61.28	−65.28	−3063	−3263

Mean individual AIC (for all participants). The group AIC is the sum of all the AIC for all group members that fit the model. One Chinese participant did not fit the Constant Sensitivity model. Another Chinese participant and one French student did not fit the Hyperbolic model. Since the vast majority of AIC values (over 97%) were negative, the non-inclusion of a group member who did not fit that model tended to penalize the group AIC of that model. Globally, all the models tended to fit the French better than the Chinese. The Constant Sensitivity model was the best model for all participants and by far the best model for the Chinese, however, the beta-delta model was the best model for the French, but the worst model for the Chinese.

**TABLE 5:** Comparison of the parameters for each social discounting model.

		Constant sensitivity		Beta delta		Hyperbolic model	
		*a*	*b*	β	δ	V	k
Chinese	Mean	0.187	0.932	0.948	0.893	1.528	0.740 1.823
	SD	0.473	0.814	0.352	0.141	1.851	
French	Mean	0.313	0.525	0.666	0.950	0.771	0.151 0.277
	SD	0.693	0.426	0.241	0.052	0.336	
P (Mann-Whitney *U*-test)		0.057	<0.001	<0.001	0.062	<0.001	0.168

Means and standard deviations for the two parameters for each of the social discounting models. Mann-Whitney *U*-tests showed the parameter b of the constant sensitivity was significantly greater for the Chinese than the French indicating a greater sensitivity to social distance, meaning that the level of generosity of the Chinese participants falls more quickly than that of the French as social distance increases. The significantly higher levels of b in the beta-delta model suggest that Chinese have greater “added value” for recipients at the closest social distances than the French, however, this must be treated with caution because this was the worst model for the Chinese. Similarly, according to the Hyperbolic model the Chinese participants show a greater level of generosity to a theoretical recipient at social distance zero, however, this was the worst model for the French.

The parameter *a*, which in our experiment delimits the border between “close” and “distant” social relations, (defined as 1/*a*), was not significantly different between French and Chinese participants (M Chinese = 0.187, M French = 0.313; *U* = 995, *Z* = 1.902, *p* = 0.057). This indicates that the social distances at which the French and Chinese participants discriminate close from distant relationships with respect to their generosity are not significantly different. However, the parameter *b* of the Constant Sensitivity model differed significantly between the Chinese and French (Mann-Whitney *U*-test; M Chinese = 0.932, M French = 0.525; *U* = 1848, *Z* = 3.892, *p* < 0.001), indicating that the Chinese show significantly greater sensitivity to social distance. This also suggests that for a significant number of Chinese participants *b* > 1. When this is the case social discounting occurs more slowly at very low social distances and then increases according to an exponential decay at the frontier between close and distant social distances as determined by the parameter *a*.

### 3.4. Regression analysis of factors affecting social discounting

**FIGURE 4:**
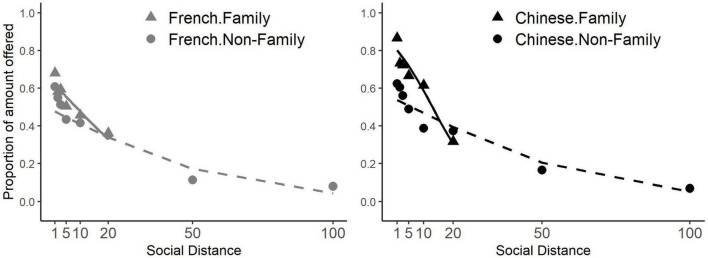
Interaction effect between group family relations and social distance on generosity at social distances 1∼100. The solid lines show the proportion of the endowments offered by participants to family members, for the French (**left** graph, blue), and the Chinese (**right** graph, red) as estimated by GLM3. The dotted lines represent the proportions of the endowments offered to non-family members. The solid triangles show the mean proportions of the endowments offered to family members for the Chinese and French. The hollow dots show the mean proportions offered to non-family members. At social distances 50 and 100 the participants did not know the recipients, who could not be family members.

To understand which factors shaped the social discounting curves of the French and Chinese participants we used General Linear Models (GLMs) to analyze the data. We conducted three GLMs for distributed differences between French and Chinese participants at social distances 1∼100. We tested whether the proportion of endowment donated by each participant was affected by the group (variable Nationality, with the French equals 1, and the Chinese equals 0), by endowment size (variable Endowment), social distance between the participant and the recipient (variable Distance), whether the recipient was a family member or not (variable Family member, equals 1 when the recipient was a family member and 0 otherwise). We ran three logit transformed GLMs and reported the marginal effects (Figure 4 and Table 6). We varied whether we included interactions between the variable Nationality, Distance and Family member (GLM2) and whether we included the 5 components of the IND-COL scale (GLM3).

**TABLE 6:** Generalized linear models predicting social discounting behavior.

	GLM 1	GLM 2	GLM 3	GLM 4	GLM 5	GLM 6
Parameters	Marginal effects (standard errors)					
French	−0.077 (0.300)**	−0.067 (0.300)**	−0.087 (0.032)***	−0.093 (0.036)**	−0.084 (0.036)**	−0.105 (0.040)**
**Endowment**	<0.001 (<0.001)***	<0.001 (< 0.001)***	<0.001 (< 0.001)***	<0.001 (0.001)***	<0.001 (<0.001)***	<0.001 (< 0.001)***
**Distance**	−0.006 (< 0.001)***	−0.011 (< 0.001)***	−0.011 (< 0.001)***	−0.015 (0.001)***	−0.015 (0.001)***	−0.015 (0.001)***
Family member	0.126 (0.176)***	0.077 (0.016)***	0.075 (0.015)***	0.122 (0.020)***	0.119 (0.020)***	0.114 (0.020)***
French × distance		0.003 (0.001)*	0.003 (0.001)*		0.004 (0.002)*	0.004 (0.002)*
French × family member		−0.060 (0.032)	−0.039 (0.033)		−0.098 (0.041)*	−0.072 (0.042)*
Family member × distance		−0.008 (0.001)***	−0.008 (0.001)***		−0.008 (0.002)***	−0.007 (0.002)**
**French × family member × distance:**						
Family member: French × distance		0.007 (< 0.001)*	0.007 (0.003)*		0.007 (0.003)*	0.007 (0.003)*
Non-family member: French × distance		<0.001 (< 0.001)	<0.001 (< 0.001)		<0.001 (< 0.001)	0.001 (0.003)
CF			0.001 (0.003)			0.001 (0.005)
PA			<0.001 (<0.001)			0.001 (0.006)
PS			0.001 (0.003)			0.001 (0.004)
KN			−0.003 (< 0.001)			−0.003 (0.005)
NE			−0.003 (0.003)			−0.004 (0.003)
Number of observations	4080	4080	4000	3060	3060	3000
AIC	0.867	0.854	0.855	0.966	0.956	0.960

Marginal effects are shown in bold type, standard errors clustered at the participants’ level are in parentheses; *p < 0.05, **p < 0.01, ***p < 0.001. We included in all GLMs the following independent variables: whether the participant was French or Chinese (variable French, Chinese = 0, French = 1), the social distance (8 level values for GLMs 1, 2 and 3:, 1, 2, 3, 5, 10, 20, 50, and 100; and 6 values for GLMs 4, 5, and 6: 1, 2, 3, 5, 10, and 20), the endowment size (€80, €90, €100, €110, €120), whether the recipient was a family member or not (variable family member). GLMs 1–3 were computed using the data over social distances 1–100, while GLMs 4–6 were computed only from social distances 1–20, in order make a more accurate comparison of how the Chinese and French might differ in their treatment of family members with non-family members at the same social distances. Standard errors clustered at the participant level are in parentheses. IND-COL results were not available for two subjects who therefore were excluded from GLM3 and GLM6, which accounts for the differences in the number of observations and p-values for GLM 3 compared to GLM1 and GLM2 and for GLM6 compared to GLM4 and GLM5. The BOLD type indicated the within-factors.

Results showed that the French were overall less generous than Chinese participants (GLM1, Nationality), that participants were more generous with larger endowments (GLM1, Endowment), that generosity decreased with increasing social distance, (GLM1, Distance) and, that participants were more generous to family members than other recipients (GLM1, Family member). This last finding indicates that both the French and Chinese family members profited from a kinship premium. Concerning the interactions reported in model GLM 2, we found that Chinese participants were more sensitive to social distance, discounting more than the French as social distance increased (GLM 2, Nationality × Distance). However, the Chinese were only marginally more generous to family members than non-family members than the French. The significance of this difference did not achieve the standard threshold of 0.05 (GLM 2, Nationality × Family member: *p* = 0.058). Social discounting was also stronger, i.e., there was a greater sensitivity to social distance for family members than non-family members, indicating that the kinship premium is severely discounted as social distance increases. We found that Chinese participants were more sensitive than the French to social distance, which was driven primarily by their treatment of family members, because this difference did not hold for non-family members (GLM2 Family member: Nationality x Distance and Non-family member: Nationality × Distance). This was further supported by the finding that the triple interaction Nationality × Family member × Distance revealed a significant difference between the French and the Chinese concerning social discounting for family members with respect to non-family members. Thus, the Chinese were significantly more sensitive than the French to the social distance of family members compared to non-family members (GLM 2). Finally, the inclusion of the five components of the IND-COL scale (GLM 3), had no significant effect on the results. This suggests that these factors do not account for any significant amount of the variance of social discounting in our participants.

The better to discriminate possible marginal effects of kinship premium or IND-COL scale factors we ran GLM 4, GLM 5 and GLM 6 which were identical to GLM 1, GLM 2, and GLM 3. However, we removed the data for social distances 50 and 100. This was justified because in our experiments, family members could not be included at social distances 50 and 100. Therefore, the inclusion of the data at these social distances obscures differences in the treatment of family and non-family members (Table 6). Indeed, when this analysis was carried out a significant interaction between Nationality × Family was revealed. Thus, over the social distances at which generosity to family members can be compared with the generosity to non-family members (Social Distances 1∼20) the Chinese showed a significantly greater generosity to family than to non-family members than the French (GLM 5). This can be appreciated from the difference between the gradients of social discounting for family as opposed to non-family members for the French and the Chinese as social distance increases (Figure 4). This was the only difference observed between GLMs 1, 2, and 3 and GLMs 4, 5, and 6. Thus, there was no significant difference between the generosity of the French and the Chinese to non-family members over social distances 1∼20 (GLM 5), and no apparent effect of the IND-COL subscales (GLM 6). Thus, the greater generosity of the Chinese was, essentially, the product of a kinship premium which resulted in Chinese participants allocating a significantly greater share of the endowments to family members especially at close social distances.

Finally, we investigated whether the parameters of the models (α and *b* for the Constant Sensitivity Model, β and δ for the Beta-Delta model, and *k* and *V* for the hyperbolic model), correlated with the individual IND-COL scores on the different subscales, on the higher order factors (Ingroup Solidarity and Social Obligation) or indeed on the global IND-COL score. No significant correlations were discovered for the Chinese students with any of the models (Supplementary Tables 1, 3, 5), or for the French with the parameters of the Beta-Delta or the Hyperbolic models (Supplementary Tables 4, 6). However, for the French with the Constant Sensitivity model (Supplementary Table 2), the parameter *a* showed a significant inverse correlation with the IND-COL subscale Kin and Neighbors/susceptibility to influence. This suggests that for French students the greater their sensitivity to the influence of family and neighbors (α measure of their integrations with society), the broader they set the boundaries of their Ingroup (1/α). The parameter *b* showed significant inverse correlations with the factors Parents and Spouse/distinctiveness of personal identity, the higher order factor Ingroup Solidarity and Global IND-COL scale. Since high scores reflect greater social integration and *b* measures sensitivity to social distance, it is entirely coherent that these factors should be inversely correlated.

## 4. Discussion

By adopting a dictator game with real recipients at each social distance, as a paradigm of the social discounting task, our study showed that the Constant Sensitivity model best accounted for social discounting in Chinese students and the Beta-Delta model was best for the French. The Chinese showed significantly greater generosity to family members at close social distances, in agreement with the concept of a strong kinship premium. At greater social distances, there was no significant difference between the generosity of the French and the Chinese. Indeed, multivariate analysis using GLMs showed there was no significant difference between the generosity of the French and the Chinese students for non-family members at any social distance. This suggests that the relative strengths of kinship premium–rather than “cultural cohesion forces”–drives the differences in social discounting between Chinese and French students at different social distances.

Cultural comparisons are a fruitful domain for behavioral research. Authors tend to agree that occidental cultures such as in North America and Western Europe tend to be more individualistic than those in Asia or Africa (Markus and Kitayama, 1991). In turn, Asian and African cultures are perceived to be more interdependent. The economic utility of generosity can be envisaged to be an investment in social capital such that today’s generosity will be repaid in some way at some time in the future. Thus, one might expect societies characterized by greater interdependence to exhibit greater generosity across the range of social distances (Archambault et al., 2020). Several studies support this by showing that although participants from China and Japan are not more generous than occidental participants, there is less difference between their generosity at low and high social distances when compared to North Americans or Germans (Strombach et al., 2014; Ishii and Eisen, 2018). This has been taken to reflect the greater integration of Asians within their culture as a whole.

A thought provoking study compared social attitudes and social discounting in college students in the USA, workers and students in China, and nomadic tribesmen in Kenya (Boyer et al., 2012). The three groups expressed similar levels of trust with respect to their social groups (family, friends, extended family, and neighbors). The Americans and Chinese showed very similar levels of generosity to others at different social distances but the Kenyans showed very little tendency to share with anyone, including close family (Boyer et al., 2012). The authors argued that this reflected the global instability of the Kenyan tribesmen’s environment where pastoral tribesmen are at the mercy of potentially devastating famines and droughts as well as aggression from neighboring tribes and intra-tribal rivalries. In such uncertain circumstances, investments in “social capital” may be unlikely to bear dividends. Similar cross cultural comparisons that have included Asian communities living in comparatively deprived environments in Indonesia and Bangladesh, have also failed to find any evidence of hyperbolic social discounting (Tiokhin et al., 2019). Instead, participants showed limited philanthropy toward other members of their communities proportional to need, rather than close social or kinship distance. These studies support that social discounting is a cultural phenomenon that is not universal and which breaks down in harsh economic conditions. However, other studies that have used social discounting tasks to assess prosocial behavior, in non-western, educated, industrialized, rich and democratic countries, reported that generosity declines across social distance, including in India (Hackman et al., 2015), Singapore (Pornpattananangkul et al., 2019), and Kenyan Massai (Archambault et al., 2020). These authors argued that the nature of the dyadic interactions, as measured by social distance, was of equal or even higher importance in these communities, because they rely on well-defined categories of social relations to allocate and access resources. Thus, they explained that the propensity for cooperation and sharing behavior varies with social distance in these societies. Individuals at close social distances are likely to directly share certain common pooled resources on a daily basis, but such day-to-day interdependencies are much less applicable with more socially remote individuals. Thus, discrepancies remain between studies to understand whether generosity depends upon social distances in different societies.

Even in the developed world and among highly educated people (e.g., university students) several issues need to be clarified with respect to social discounting. The cross-cultural studies have often used either partially or totally abstract games to compare generosity at different social distances, and have been based on binary decision choices. Therefore, we reinvestigated this issue using a non-hypothetical, non-deceptive experimental design in which the participants and recipients from their social entourage would enjoy the benefits of the participants’ decisions. For greater social distances (50 and 100), the beneficiaries were anonymous and therefore, as the participants were informed, the money was donated to a charity. The participants chose freely how to divide the endowments, so their generosity could be measured precisely.

Unlike most previous studies the best models to account for the data were the Constant Sensitivity model for the Chinese students and the Beta-Delta model for the French (Table 4 and Figure 3). Interestingly, in a previous study of social discounting by Chinese students the model with the lowest AIC was also found to be the Constant Sensitivity model (Strombach et al., 2014). We found the value of *b*, which measures the sensitivity to social distance, was significantly higher (*p* < 0.001) for the Chinese (0.932 ± 0.814) than the French (0.526 ± 0.426). This indicates that their generosity was more sensitive to social distance, which resulted in a swifter decline in generosity as social distance increased. The second parameter, *a*, defines the limit at which participants separate close from distant relationships. When *b* is greater than 1, which would appear to be the case for nearly 50% of Chinese participants, social discounting for close individuals (those at social distances less than 1/*a*) occurs more slowly than when the social distance increases to greater than 1/a. This results in an initial plateau of high generosity to very close individuals. Such behavior is contrary to the hyperbolic and the beta-delta models and would explain why the constant sensitivity model is by far the best for the Chinese participants. The value of *a* was not significantly different between our groups of Chinese and French students, which suggests that both groups set this distance somewhere between social distance 3 and 5. Interestingly, this also corresponded to the social distance at which the Chinese students ceased to be more generous than the French, and at which both groups, but more particularly the Chinese, ceased to choose a majority of family members as recipients. This pattern is in general agreement with the concept of a kinship premium for generosity (Curry et al., 2013; Booysen et al., 2018a). At social distances greater than 5 the French designated significantly larger numbers of family members than the Chinese. It is interesting that these family members were designated outside the boundary of close social relationships, something significantly rarer for the family members of Chinese students. Moreover, our results indicated that the Chinese were significantly more generous than the French, but only at close social distances, and only to family members.

We conducted a regression analysis to explore the sources of the differences between the social discounting of the Chinese and French students. At the first level of analysis (without exploring interactions between variables), the Chinese participants were more generous than the French, participants were more generous to recipients at closer social distances, and participants were more generous to family than non-family members. However, analysis of interactions between these variables (GLM 2, GLM 5) revealed interesting differences between the giving behavior of the Chinese and French. The significant interaction between group and social distance confirmed that the Chinese were more sensitive to social distance than the French, as suggested by the comparison of the parameter *b* of the Constant Sensitivity model. Thus, although at close social distances the Chinese were more generous than the French, as social distance increased, the difference in generosity decreased between the Chinese and the French. The motor for this difference appeared to be a strong effect of family membership, since the difference in generosity between the Chinese and the French did not hold for non-family members. However, the interaction between Group and Family membership, which directly compares the difference in generosity of the Chinese and French for their family members vs. non-family members at the different social distances, failed to attain significance when all social distances were included in the analysis (GLM 2). We reasoned that this result might be explained by the fact that at social distances 50 and 100 there could be no difference in the generosity of the French and Chinese between family and non-family members because no family members could be allocated to these social distances. Thus, the inclusion of these social distances in the analysis might potentially obscure a genuine effect of family vs. non-family membership between the French and Chinese. Therefore, we conducted GLMs 4, 5, and 6 in which we included only the data for social distances 1 to 20. As a result, we identified that the difference in generosity to family as opposed to non-family members, at the same social distance, was significantly greater for the Chinese than for the French, especially at the closest social distances. That is to say that the kinship premium of the Chinese is significantly larger than that of the French. Furthermore, there was no apparent difference between the generosity of the French and the Chinese for non-family members at any social distance. Interestingly, the kinship premium was especially sensitive to social distance, and even more so among the Chinese than the French participants, as shown by the results of the Nationality × Family member × distance triple interaction (GLM 2, GLM 5).

The results of the IND-COL scales seemed to predict results that were very different than those obtained for social discounting. Firstly, the scores for Ingroup Solidarity, a measure of the closeness of the relationship between the participants and their closest social elements, was not significantly different between the French and Chinese and therefore, might be expected to predict similar levels of generosity at close social distances. This idea was reinforced by the results of the three individual subscales that contribute to this higher order scale (CF, PS, and PA), none of which showed significant differences between the French and Chinese. However, in the Social Discounting experiments the Chinese were significantly more generous than the French at close social distances. The Social Obligation scale, as well as the two subscales that contribute to it (KN and NE), indicated that the Chinese were significantly more integrated and collective with more distant elements of their social entourage, and their society as a whole. This might predict that the Chinese should be expected to show higher levels of generosity at greater social distances than the French, however the Social Discounting task revealed no such differences.

In a previous study (Booysen et al., 2021), within subject correlations occurred between one of the parameters of the hyperbolic social discounting model (*k*) and social collectivism or individualism as measured by an IND-COL test, such that more individualistic participants were more sensitive to social distance. We therefore analyzed for correlations between the different model parameters. For the Chinese participants, no significant correlations were found. For the French, inverse correlations between parameters α and *b* in the Constant Sensitivity model and the scores on several of the IND-COL subscales and the Global IND-COL scores were coherent. Thus, French participants with higher scores for social integration set the limits between close and distant relationships at greater social distances from themselves (lower α) and were less sensitive to social distance (lower *b*). However, the results of GLM 3 and GLM 6 surprisingly showed that IND-COL subscale scores had no significant capacity to explain the variance of social discounting among the participants. There is a crucial difference between the two types of experiments. In the IND-COL scale, statements by the participant with regard to their social preferences are cost free. Similarly, in the Self-Representation task the estimation of the social distance to others in one’s social entourage has no price and no payoff. In our social discounting task, the participants lose money from their own payoff in order to give money to others at each social distance. How much they are “willing to pay” to make that gift provides a real measure of the value of their generosity to that person. Many experiments on social discounting have been performed using either completely hypothetical payoffs or asymmetrical payoffs that only affected the participant, i.e., the payoffs are made to the participant but not the recipient. In such a case, when participants decide to allocate more of the endowment to a hypothetical recipient, they are essentially paying for the pleasure of making a purely hypothetical gift. A decision that *homo economicus* should reject. This may well impinge on the levels of generosity, especially at close social distances, when the cost might be proportionally higher, and hence result in lower estimations of the kinship premiums at close social distances. Perhaps it is this difference, the fact that one must pay a price to express a higher degree of social integration in the social discounting task, that accounts for the lack of correlation between the degree of social integration expressed in the IND-COL, the Self Representation task and the Social Discounting tasks.

The principal difference we found between the Chinese and French students concerns a significantly greater kinship premium for the Chinese than the French. Family and kinship are central to both the Occidental and Chinese cultures, however, it may be that the family, its stability and the importance of family relationships are greater in China than in France. This might be reflected by national statistics. The number of divorces per 100 marriages in France in 2016 was estimated at 55% (Eurostat, 2021), compared to only 3% for China (Ministry of Civil Affairs of the People’s Republic of China, 2017). This is also reflected by the fact that 26% of families in France are single parent families compared to 10% in China. These data suggest that the family unit in China is indeed more stable. For example, the majority (almost 60%) of live births in France in 2016 were to unmarried parents (Eurostat, 2018), a statistic we did not find for China. This suggests that the majority of French parents no longer necessarily get married. These data all suggest that formal family relationships may be less concrete in France than China.

This impression is also reinforced by the results of our Self-Representation task. The French placed Partners (girlfriend/boyfriend) most frequently as the closest person in their entourage, with mothers second and fathers at a distant fifth position. In contrast the Chinese placed mothers and fathers similarly close at first and second positions. Although, the mean social distance at which Chinese participants placed their “partners” was not significantly greater than that of the French, they placed four other categories (mother, father, brothers and sisters, and best friend) at closer mean social distances. It is interesting that all the French participants indicated they had a partner, whereas only 45% of the Chinese did so. This might suggest that the French were including more trivial relationships as “Partners,” however, the fact that on average, this was perceived as the closest relationship the participants had, suggests this was not the case.

The greater relative closeness of family members, the stability and the apparent importance of the family unit to the Chinese participants all fit well with the higher magnitude of their kinship premium. As the social distance increases, the value of this kinship premium was discounted rapidly, for both the French and the Chinese, but especially for the Chinese. Furthermore, the proportion of family members falls as social distance increases, and these combined effects result in the increased sensitivity to social distance of the Chinese compared to the French.

## Data availability statement

The original contributions presented in this study are included in the article/Supplementary material, further inquiries can be directed to the corresponding authors.

## Ethics statement

Ethical review and approval was not required for the study on human participants in accordance with the local legislation and institutional requirements. The patients/participants provided their written informed consent to participate in this study.

## Author contributions

JL: conceptualization, methodology, formal analysis, data analysis, and writing. ED, CQ, and J-CD: conceptualization and writing. JB: data analysis and writing. BC and J-BV: writing—review and editing. ZT: conceptualization. All authors provided the critical revisions.
