# Supplementary material for: Cross-cultural study of kinship premium and social discounting of generosity
Source: Front Psychol. 2023 Feb 24;14:1087979. doi: 10.3389/fpsyg.2023.1087979 (PMC10000291; doi:10.3389/fpsyg.2023.1087979)
Supplement: Supplementary file 1 [file Data_Sheet_1.doc]

# Cross-cultural study of kinship premium and Social Discounting of Altruism

## Supplementary Material

**Instructions for the Experiment (translated from Chinese)**

Thank you for participating in this experiment on decision making and Social Distance.

## Stage 1: Self-representation Task

Social distance means the inter-personal, psychological distance between us and people in our daily lives, commonly known as closeness or familiarity. In the table below (Self-Representation Task), you need to indicate the social distance between you and the following people from 1 to 100. Social distance 1 is the closest social distance possible, while Social distance 100 means the furthest social distance, like for a complete stranger.

1. Please rate your perceived closeness to specific people in your social environment on a 100-point scale.

| mother | father | siblings | grand-  parents | kin | best friend | roommate |
| --- | --- | --- | --- | --- | --- | --- |
|  |  |  |  |  |  |  |
| colleagues | neighbors | acquaintances | lover | stranger | circle of friends | |
|  |  |  |  |  |  | |

## **Stage 2: Social Distance Relationship Formula**

Please imagine your social network has 100 social distances, social distance 1 is the closest person in your real life. The larger the social distance number, the greater the social distance. We define social distance 100 as a complete stranger, and social distance 50 as a person you have seen before, but you don’t know anything about them at all. Here, you need to complete this table by writing down the name, relationship, and a contact address for the people who best represent the 8 social distances from you. What you should be careful of is that if, for example, there are 5 people that could represent social distance 1, you should choose only one of them for social distance 1. You should not put their names at the other social distance levels from 1 to 5.

1. Social Distance Formula.

| social distance | relationship | name | contact address (phone, e-mail, etc.) |
| --- | --- | --- | --- |
| 1 |  |  |  |
| 2 |  |  |  |
| 3 |  |  |  |
| 5 |  |  |  |
| 10 |  |  |  |
| 20 |  |  |  |
| 50 | The person you have seen, but you do not know |  |  |
| 100 | stranger |  |  |

## **Stage 3: Social discounting task**

For the following experiment, you need to keep in mind the people corresponding to each social distance that you indicated in the form. If you don’t remember who they are, you can check them on the list anytime. This task has 40 trials presented randomly, in which you have to share 5 different endowments (¥80/90/100/110/120) between you yourself and the recipients at 8 social distances. It will start with a white "+" in the centre of the computer screen to remind you to start the experiment and pay attention.

Next, at the top of screen, you will see a white icon at the left end of the scale that represents you, and a blue icon with given social distance number, that stands for the person “**A**” that you indicated for that social distance in the form you filled in. Next, the screen will show an initial endowment of money which belongs to both of you. However, you are the allocator and you can divide the money between the two of you as you choose. The person “**A”** is the receiver who has to accept whatever you decide. You indicate the amount that you are willing to give **A**, by typing it in the box and pressing “Enter” to confirm. You should Notes that you cannot give “A” more than the total amount. Finally, you will see the feedback that shows how much money you and “A” each received.

At the end of the experiment one of these trials will be chosen at random by the computer and rewarded at 10% of the selected total amount. The money will be given to you and the receiver, whoever they are. If the selected trial involves a recipient at social distance 50 or 100, we will send the money to an online charity project.

After you understand the instructions above, please press the button ‘P’ to practice (5 training trials).

## **Stage 4: Individualism/Collectivism Questionnaire**

Please complete this questionnaire, to finish the experiment.

The questionnaire includes Individualism-Collectivism scale and the two questions below：

4.1. Which three words best describe the Chinese/French (as appropriate) culture for you?

4.2. To what extent do you personally identify with the Chinese/French (as appropriate) culture on a scale of 1 to 5, where 1 indicates that you do not identify with it at all and 5 indicates that you identify with it completely.

## Supplementary data

## Table S1 & S2: Pearson correlation between IND-COL sub-scales, higher order factors and global scores and parameters *a* and *b* of Constant Sensitivity model.

**Chinese**


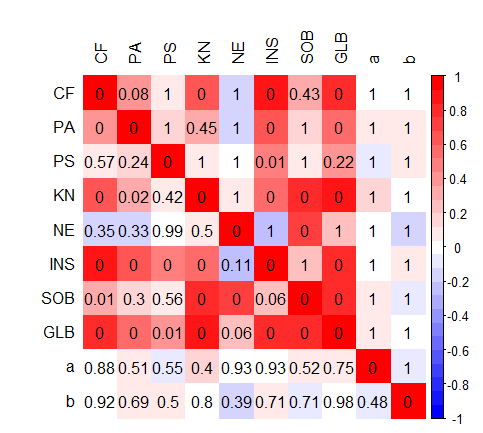


**French**


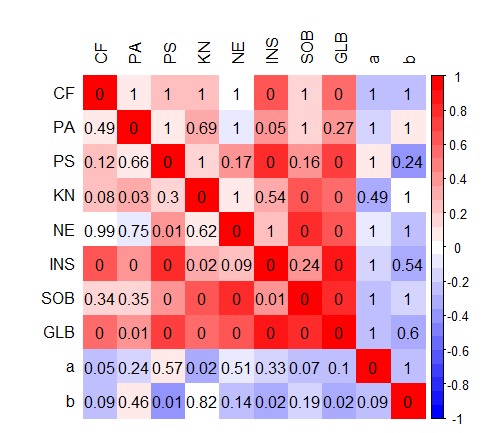


Notes: correlations are shown in red boxes and inverse correlations in blue, together with the levels of significance (p value) according to the Pearson correlation. CF, PA, PS, KN and NE stand for subscales Colleagues and Friends/supportive exchanges, Parents/consultation and sharing, Parents and Spouse/distinctiveness of personal identity, Kin and neighbors/susceptibility to influence, and Neighbor/social isolation respectively. INS, SOB and GLB stand for the Ingroup Solidarity, Social Obligation higher order factors and the global IND-COL score.

## Table S3 & S4: Pearson correlation between IND-COL sub-scales, higher order factors and global scores and parameters β and δ of Beta-delta model.

**Chinese**


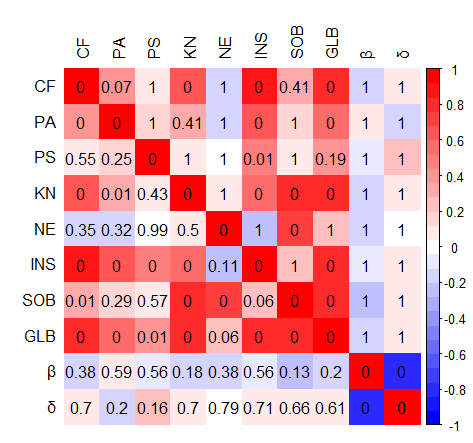


**French**


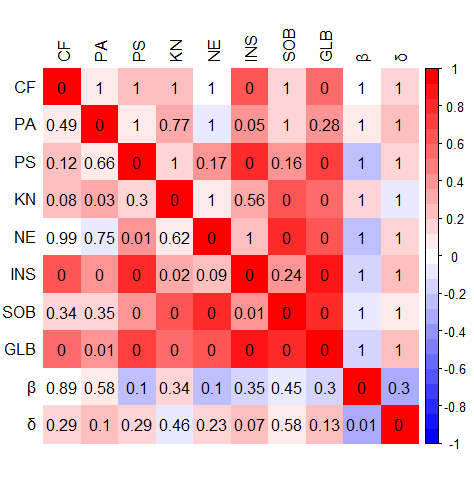


Notes: correlations are shown in red boxes and inverse correlations in blue, together with the levels of significance (p value) according to the Pearson correlation. CF, PA, PS, KN and NE stand for subscales Colleagues and Friends/supportive exchanges, Parents/consultation and sharing, Parents and Spouse/distinctiveness of personal identity, Kin and neighbors/susceptibility to influence, and Neighbor/social isolation respectively. INS, SOB and GLB stand for the Ingroup Solidarity, Social Obligation higher order factors and the global IND-COL score.

## Table S5 & S6: Pearson correlation between INCOM scale and parameters V and k of Hyperbolic model：

**Chinese**


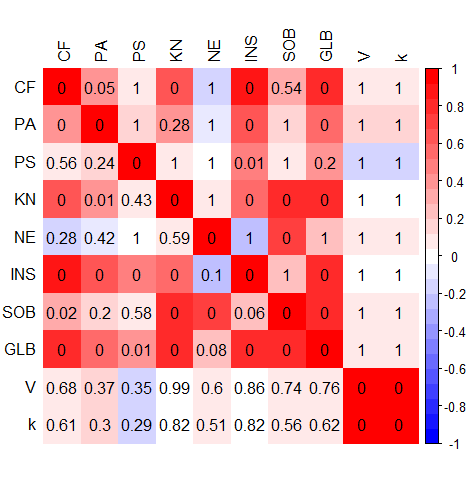


**French**


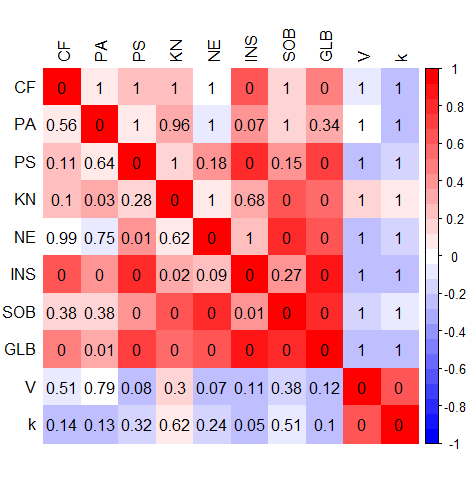


Notes: correlations are shown in red boxes and inverse correlations in blue, together with the levels of significance (p value) according to the Pearson correlation. CF, PA, PS, KN and NE stand for subscales Colleagues and Friends/supportive exchanges, Parents/consultation and sharing, Parents and Spouse/distinctiveness of personal identity, Kin and neighbors/susceptibility to influence, and Neighbor/social isolation respectively. INS, SOB and GLB stand for the Ingroup Solidarity, Social Obligation higher order factors and the global IND-COL score.
